# TRPM4 Contributes to Subthreshold Membrane Potential Oscillations in Multiple Mouse Pacemaker Neurons

**DOI:** 10.1523/ENEURO.0212-21.2021

**Published:** 2021-11-12

**Authors:** Keyong Li, Yingtang Shi, Elizabeth C. Gonye, Douglas A. Bayliss

**Affiliations:** Department of Pharmacology, University of Virginia, Charlottesville, VA 22908

**Keywords:** calcium-dependent, cationic current, ICaN, tonic firing, TRP channels, waveform command

## Abstract

Select neuronal populations display steady rhythmic neuronal firing that provides tonic excitation to drive downstream networks and behaviors. In noradrenergic neurons of the locus coeruleus (LC), circadian neurons of the suprachiasmatic nucleus (SCN), and CO_2_/H^+^-activated neurons of the brainstem retrotrapezoid nucleus (RTN), large subthreshold membrane potential oscillations contribute to the pacemaker-like action potential discharge. The oscillations and firing in LC and SCN involve contributions from leak sodium (NALCN) and L-type calcium channels while recent work from RTN suggested an additional pivotal role for a secondary calcium-activated and voltage-gated cationic current sensitive to TRPM4 channel blockers. Here, we tested whether TRPM4 contributes to subthreshold oscillations in mouse LC and SCN. By RNAscope *in situ* hybridization, *Trpm4* transcripts were detected in both cell groups. In whole-cell recordings from acute slice preparations, prominent voltage-dependent membrane potential oscillations were revealed in LC and SCN after blocking action potentials. These oscillations were inhibited by two chemically-distinct blockers of TRPM4, 9-phenanthrol (9-pt) and 4-chloro-2-[[2-(2-chlorophenoxy)acetyl]amino]benzoic acid (CBA). Under whole-cell voltage clamp, inward currents evoked by oscillation voltage waveforms were inhibited in LC by blocking L-type calcium channels and TRPM4. These data implicate TRPM4 in the large subthreshold membrane potential oscillations that underlie tonic action potential discharge in LC and SCN, providing a voltage-dependent and calcium-dependent cationic current to augment the depolarizing inward Na^+^ and Ca^2+^ currents previously associated with this distinctive electroresponsive property.

## Significance Statement

Large subthreshold membrane potential oscillations contribute to spontaneous action potential discharge in select neurons, including those of the locus coeruleus (LC) and the suprachiasmatic nucleus (SCN). The ionic mechanisms underlying these intrinsic membrane properties are incompletely understood. The present study identifies a role for the calcium-activated TRPM4 cation channel in mediating subthreshold oscillations and firing in LC and SCN, supporting previously described roles for NALCN leak sodium channels and L-type calcium channels while generalizing this TRPM4-dependent oscillatory mechanism to multiple pacemaker-type neurons.

## Introduction

The ability to fire action potentials in a regular pacemaker-like pattern is an important intrinsic property of various types of neurons that can, for example, provide tonic excitatory or inhibitory drive to downstream circuits, or a baseline activity level as comparator for dynamic modulation. This regular activity can be sustained in different cell types by a variety of interacting membrane ion channel currents that ultimately determine the firing pattern (Häusser et al., 2004; Bean, 2007). Of particular interest, a large subthreshold membrane potential oscillation has been uncovered that can drive spontaneous action potential discharge. These voltage-dependent oscillations are revealed after blocking fast voltage-activated sodium (Na_V_) channels, and are typically ∼20 mV in amplitude in the 2- to 4-Hz frequency range. Specifically, they have been observed in suprachiasmatic nucleus (SCN) neurons that comprise the circadian clock (Pennartz et al., 2002; Jackson et al., 2004), dopaminergic neurons of the substantia nigra pars compacta (SNpc; Yung et al., 1991; Nedergaard and Greenfield, 1992; Nedergaard et al., 1993; Chan et al., 2007; Puopolo et al., 2007), noradrenergic neurons of the locus coeruleus (LC; Christie et al., 1989; Filosa and Putnam, 2003; Imber and Putnam, 2012; Sanchez-Padilla et al., 2014), and most recently in *Nmb*-expressing respiratory chemoreceptor neurons in the retrotrapezoid nucleus (RTN; Li et al., 2021).

In terms of ion channel pharmacology, it was recently demonstrated that subthreshold oscillations in RTN neurons depends on both L-type calcium channels (i.e., they were sensitive to dihydropyridine antagonists) and on TRPM4 calcium-dependent cationic channels [i.e., they were sensitive to 9-phenanthrol (9-pt) and 4-chloro-2-[[2-(2-chlorophenoxy)acetyl]amino]benzoic acid (CBA); Li et al., 2021]. The seemingly similar oscillations in SCN, SNpc, and LC neurons also include contributions from L-type calcium channels but a role for TRPM4 channels has not yet been evaluated (Yung et al., 1991; Nedergaard and Greenfield, 1992; Nedergaard et al., 1993; Pennartz et al., 2002; Filosa and Putnam, 2003; Jackson et al., 2004; Chan et al., 2007; Puopolo et al., 2007; Sanchez-Padilla et al., 2014; Flourakis et al., 2015). Here, we used *in situ* hybridization, patch clamp recording and ion channel pharmacology to demonstrate that *Trpm4* is expressed in LC and SCN neurons, and to show that TRPM4 contributes to subthreshold oscillations observed in those cells in neonatal mouse brainstem slices. The TRPM4 current evoked by oscillatory waveform command was particularly sensitive to nifedipine in LC neurons, consistent with a privileged role for L-type channels in supporting the calcium-dependent and voltage-dependent TRPM4 cationic current.

## Materials and Methods

### Animals

Experiments were performed on mice of either sex, following procedures adhering to National Institutes of Health *Animal Care and Use Guidelines* and approved by the Institutional Animal Care and Use Committee. Mice were housed in Allentown Caging HEPA ventilated racks and steam-sterilized caging (up to four per cage), with crushed corncob bedding and *ad libitum* access to irradiated Teklad diet (7904 or 7912) and reverse osmosis water provided through an Avidity automatic drinking water system. Animals were exposed to 12/12 h light/dark cycles in a vivarium maintained at 22–24°C and ∼40% relative humidity. We used a Phox2b::GFP BAC transgenic mouse line (Jx99) that was developed by the GENSAT project and characterized previously (Lazarenko et al., 2009). A total of 67 mice were used (57 neonates for *in vitro* electrophysiological recording; five adults and five neonates for *in situ* hybridization experiments).

### Mouse brainstem slice preparation

Brain slices were prepared from neonatal Phox2b::GFP mice at ∼4 h after lights on at 7 A.M. [i.e., at ∼11 A.M., around zeitgeber time (ZT)4; Li et al., 2021]. Briefly, pups [postnatal day (P)6–P14] were anesthetized with ketamine and xylazine (375 and 25 mg/kg, i.m.), rapidly decapitated, and brain slices (300 μm) were cut through the LC and SCN regions in ice-cold sucrose-substituted solution, containing the following: 260 mm sucrose, 3 mm KCl, 5 mm MgCl_2_, 1 mm CaCl_2_, 1.25 mm NaH_2_PO_4_, 26 mm NaHCO_3_, 10 mm D-glucose, and 1 mm kynurenic acid. Slices were incubated for 30 min at 37°C and subsequently at room temperature in normal Ringer’s solution containing the following: 130 mm NaCl, 3 mm KCl, 2 mm MgCl_2_, 2 mm CaCl_2_, 1.25 mm NaH_2_PO_4_, 26 mm NaHCO_3_, and 10 mm D-glucose. Both cutting and incubation solutions were bubbled with 95% O_2_ and 5% CO_2_.

### *In vitro* electrophysiology

Electrophysiological recordings were obtained from ZT5.5 to ZT10 in brain slices from GFP-labeled LC neurons located in the rostral brainstem at the lateral edges of the fourth ventricle or visually identified SCN neurons adjacent to the third ventricle and above the optic chiasm. LC and SCN neurons were recorded at room temperature, a condition that better preserves the health of the slice. We obtained recordings in cell-attached or whole-cell configuration using pClamp, an Axopatch 200B amplifier, and a Digidata 1322A analog-to-digital converter (all from Molecular Devices). For recording, the brainstem slices were placed in a recording chamber mounted on a fluorescence microscope (Zeiss Axioskop FS) in HEPES-based perfusate, containing the following: 140 mm NaCl, 3 mm KCl, 2 mm MgCl_2_, 2 mm CaCl_2_, 10 mm HEPES, and 10 mm D-glucose. For whole-cell current-clamp recordings or cell-attached voltage-clamp recordings, pipettes were filled with an internal solution containing the following: 17.5 mm KCl, 112.5 mm K-gluconate, 1.5 mm NaCl, 5 mm Na_2_phosphocreatine, 1 mm MgCl_2_, 10 mm HEPES, 0.2 mm EGTA, 3 mm Mg-ATP, and 0.3 mm GTP-Tris (pH 7.2, with KOH). Voltage-clamp recordings of currents evoked by oscillatory waveforms were obtained using a Cs^+^-based internal solution containing the following: 100 mm CsCH_3_SO_3_, 1 mm MgCl_2_, 0.5 mm CaCl_2_, 5 mm Na_2_phosphocreatine, 30 mm TEACl, 10 mm HEPES, 10 mm EGTA, 3 mm Mg-ATP, and 0.3 mm GTP-Tris (pH 7.2, with CsOH). When filled with these solutions, patch electrodes had a DC resistance of 3–6 MΩ; electrode tips were coated with Sylgard 184 (Dow Corning). All reported membrane potentials were corrected for liquid junction potentials measured between the bath and these internal solutions (both ∼10 mV).

In all experiments, a cocktail of blockers was added (10 μm CNQX, 10 μm bicuculline, and 30 μm strychnine) to inhibit fast excitatory (glutamate) and inhibitory transmitters (GABA, glycine), and subthreshold oscillations were recorded after blocking tetrodotoxin (TTX)-sensitive Na_V_ channels with TTX (0.5 μm). We added CdCl_2_ (200 μm) to the bath to block Ca^2+^ currents nonspecifically and we applied nifedipine (10 μm) to inhibit L-type calcium currents. We used either 9-pt (30 μm) or CBA (50 μm) as selective TRPM4 channel inhibitors.

After obtaining high-resistance seals (>1 GΩ), cell-attached recordings of spontaneous firing behavior were obtained under voltage clamp at Vhold = −60 mV; the small interspike pipette current at this holding potential (typically less than ±4 pA) was stable throughout the recordings but may have influenced the baseline spontaneous firing rate measured in LC and SCN neurons (Perkins, 2006). Whole-cell current-clamp recordings of action potentials and subthreshold oscillations were recorded at different membrane potentials achieved by DC current injection. Subthreshold oscillations (30 s) were analyzed by fast Fourier transform (FFT; MATLAB). The power spectral density (PSD) was computed with the MATLAB function “periodogram” for each frequency bin (0.03 Hz), and total power calculated from the integrated area of the full power density spectrum. The mean frequency was calculated by MATLAB function “meanfreq.” Membrane potential oscillations were captured under current clamp and used as voltage clamp commands to characterize contributions of select currents to the oscillation-waveform trajectory under whole-cell voltage clamp. The blocker-sensitive currents were obtained by digital subtraction, and the total charge attributed to different currents assessed by integrating the blocker-sensitive currents versus time.

### Labeling recorded cells with Lucifer yellow

Recording pipettes for cell attached recording contained 0.02% Lucifer yellow. After cell attached recordings were completed, negative pressure was applied to the recording pipette to rupture the membrane. Cells were held in the whole-cell configuration for 3–5 min to allow exchange of internal solution into the cell, and then a slight amount of positive pressure was applied to the pipette to detach it from the cell. Slices were then placed in 4% paraformaldehyde (PFA) in phosphate buffer (PB; 0.1 m sodium phosphate) and incubated overnight at 4°C. After fixation, slices were washed at room temperature in PB (3 × 5 min), and in Tris saline (TS) buffer (3 × 5 min) and blocked for 45 min in 10% normal horse serum (NHS)/0.3% Triton X-100/TS. After blocking, slices were incubated with primary antibody (rabbit anti-Lucifer yellow, 1:1000; Thermo Fisher Scientific A5750, RRID:AB_2536190) in 1% NHS/0.1% Triton X-100/TS for 18 h at 4°C. Slices were washed in TS (3 × 10 min) and then incubated with secondary antibody (donkey anti-rabbit Cy3, 1:500; Jackson ImmunoResearch 711-165-152, RRID: AB_2307443) in TS for 90 min at room temperature. Slices were washed in TS (3 × 10 min) and mounted on a charged microscope slide (Fisher 12-550-15) and dried completely before coverslipping with Prolong Gold antifade mounting media containing DAPI (ThermoFisher P36935).

### Multiplex *in situ* hybridization and immunohistochemistry

Mice were perfused transcardially with 4% PFA/0.1 m PB. Brains were removed, immersed in the same fixative for 16–18 h at 4°C, cut in the transverse plane (30 μm) and placed in cryoprotectant (30% ethylene glycol, 20% glycerol, and 50 mm sodium PB, pH 7.4) at −20°C until further processing. Sections were washed in sterile PBS, mounted onto charged slides, and dried overnight. Multiplex *in situ* hybridization to combine *Trpm4* mRNA labeling with detection of either *Th*, *Avp*, or *Vip* transcripts was performed by RNAscope, following manufacturer’s instructions [Advanced Cell Diagnostics (ACD): RRID:SCR_012481]. Sections were incubated in hydrogen peroxide (10 min, 24°C), and then in target retrieval solution (5 min, 98–102°C), rinsed in sterile water (x 2), dehydrated in 100% EtOH (5 min), and dried before incubation in protease IV (30 min, 40°C). After rinsing in sterile water (2×), sections were incubated with RNAscope catalog oligonucleotide probes for *Trpm4*, *Th*, *Avp*, or *Vip* (2 h, 40°C), and processed using the ACD Multiplex Fluorescent Reagent kit v2. To combine *in situ* hybridization for *Th* with immunostaining for GFP, sections were processed for *Th* labeling as above, using the v1 version ACD Fluorescent Multiplex Detection reagents. Sections were subsequently immunochemically stained for GFP. Briefly, sections were incubated in blocking buffer (10 min; 10% NHS, 0.1% Triton X-100 diluted in PBS), in primary antibody (1 h; chicken anti-GFP, Aves Laboratories, GFP-1020), washed twice with PBS, and then incubated in the secondary antibody (30 min; donkey anti-chicken Alexa Fluor 488, Jackson ImmunoResearch, 703-545-155). Sections were washed twice with RNAscope wash buffer, air dried and covered with Prolong Gold with DAPI anti-fade mounting medium (Invitrogen).

### Statistics

Results are analyzed and presented using estimation statistics (Bernard, 2019; Calin-Jageman and Cumming, 2019), as described in respective figure legends. Source data for figures are provided in Extended Data 1.

10.1523/ENEURO.0212-21.2021.ed1 Extended Data 1 Source data and analysis for data plotted in figures. Download Extended Data 1, XLS file.

## Results

### A prominent subthreshold oscillation in LC neurons

**Figure 1.**
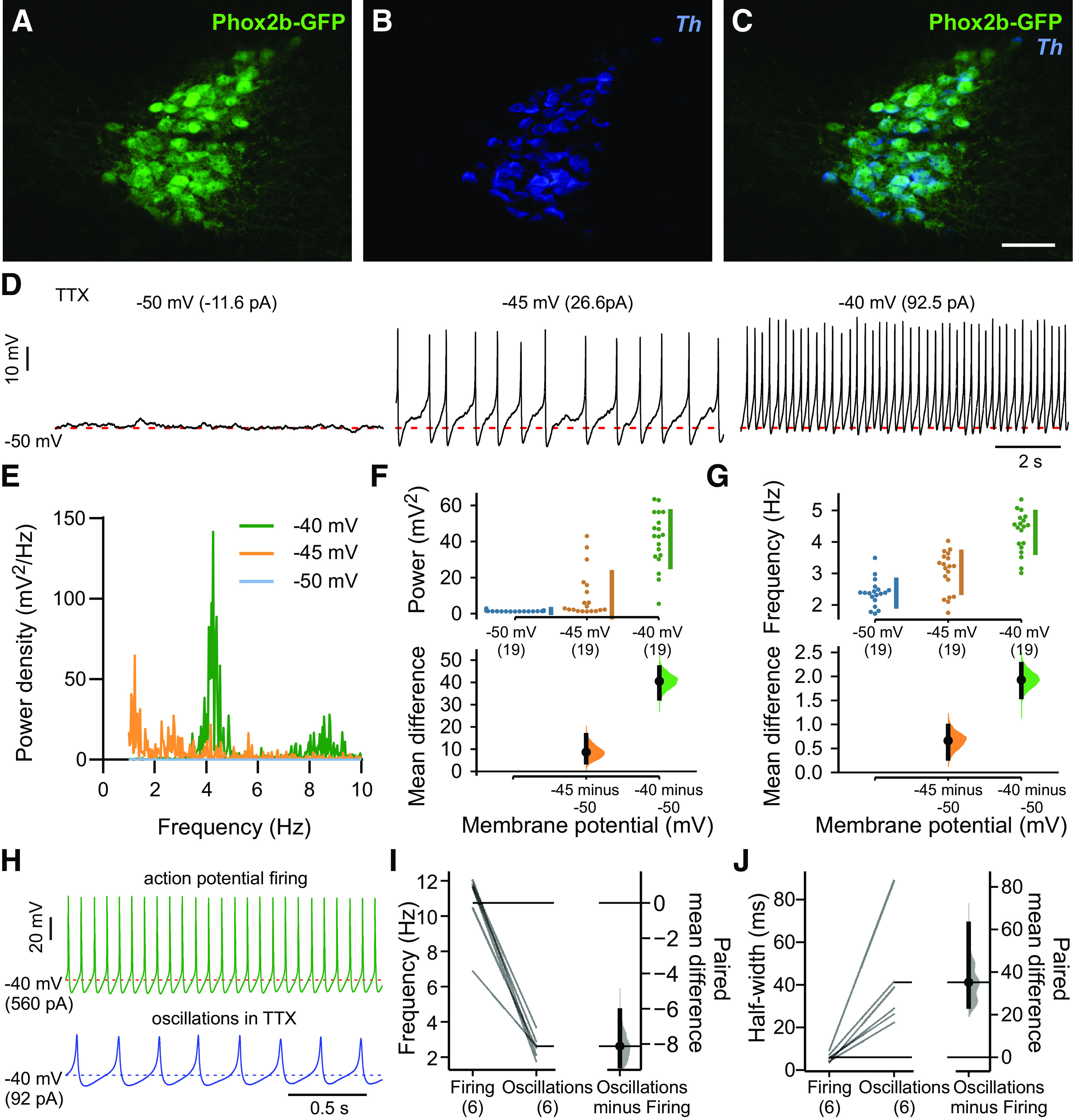
Subthreshold oscillations in LC neurons. ***A–C***, Combined immunohistochemistry for GFP and *in situ* hybridization for *Th* in the LC of a Phox2b::GFP mouse. Scale bar: 100 μm. ***D***, Small membrane potential fluctuations at −50 mV were transformed into large subthreshold oscillations when DC current was injected to depolarize the interoscillation membrane potential. Except where noted, the bath solution in this and all experiments contained TTX (0.5 μm) along with fast synaptic blockers (10 μm CNQX, 10 μm bicuculline, 30 μm strychnine). ***E–G***, Power density-frequency plots for subthreshold oscillations at the indicated membrane potentials were obtained by FFT (***E***), and integrated power (***F***) and mean frequency of subthreshold oscillations (***G***) were plotted at three membrane potentials in the same neurons (*n* = 19). The mean differences from −50 mV are presented on the lower plots as bootstrap sampling distributions, with mean depicted as a dot and 95% confidence intervals as vertical error bars. Data were assessed statistically by two-sided permutation *t* test (***F***, *p* < 0.0001 for −45 and −40 mV; ***G***, *p* = 0.0008 for −45 mV and *p* < 0.0001 for −40 mV). ***H***, Exemplar action potentials and subthreshold oscillations recorded in the same neuron at −40 mV (by DC current injection). ***I***, ***J***, Summary data (*n* = 6) depicting the lower frequency (***I***) and longer duration (half-width, ***J***) of subthreshold oscillations, relative to spontaneous firing. The paired mean differences are presented as bootstrap sampling distributions for firing frequency (***I***, *p* = 0.03) and for half-width (***J***, *p* < 0.0001), analyzed by two-sided permutation *t* test.

In Phox2b::GFP mice, the noradrenergic LC can be identified by its location in the rostral brainstem at the lateral edges of the fourth ventricle (from ∼5.34 to 5.68 mm caudal to bregma; Paxinos and Franklin, 2001), and by neuronal co-expression of tyrosine hydroxylase (*Th*) and GFP (Fig. 1*A–C*). In brainstem slice preparations from Phox2b::GFP mice, whole-cell current-clamp recordings in GFP-expressing LC neurons revealed a characteristic tonic action potential discharge under conditions where fast synaptic input is blocked (Li and Putnam, 2013). After eliminating action potential firing with TTX (0.5 μm), a large voltage-dependent subthreshold oscillation was revealed when DC current injection was used to progressively depolarize the membrane potential beyond −50 mV (Fig. 1*D*). The oscillations were analyzed by FFT in individual LC neurons at membrane potentials from −50 to −40 mV (*n* = 19), with the strongest frequency components appearing in the 2- to 4-Hz band across that voltage range (Fig. 1*E*). The subthreshold oscillations increased in power and frequency at more depolarized potentials (Fig. 1*F*,*G*), and consistently occurred at lower frequencies and with longer durations than the corresponding action potentials recorded in the same cells before TTX (Fig. 1*H–J*). The properties of these depolarization-evoked subthreshold oscillations are similar to those described previously in LC neurons, where they were variously called TTX-insensitive spikes (Filosa and Putnam, 2003; Imber and Putnam, 2012) or “spikelets” (Sanchez-Padilla et al., 2014).

### TRPM4 channels underlie subthreshold oscillations in LC neurons

A similar subthreshold oscillation in RTN chemosensory neurons requires activity of L-type calcium channels and, secondarily, the calcium-activated TRPM4 nonspecific cation channel (Li et al., 2021). In previous work from LC neurons, the subthreshold oscillation (i.e., TTX-insensitive spikes and spikelets) was also attributed to L-type calcium channels but involvement of TRPM4 was not assessed (Filosa and Putnam, 2003; Imber and Putnam, 2012; Sanchez-Padilla et al., 2014).

**Figure 2.**
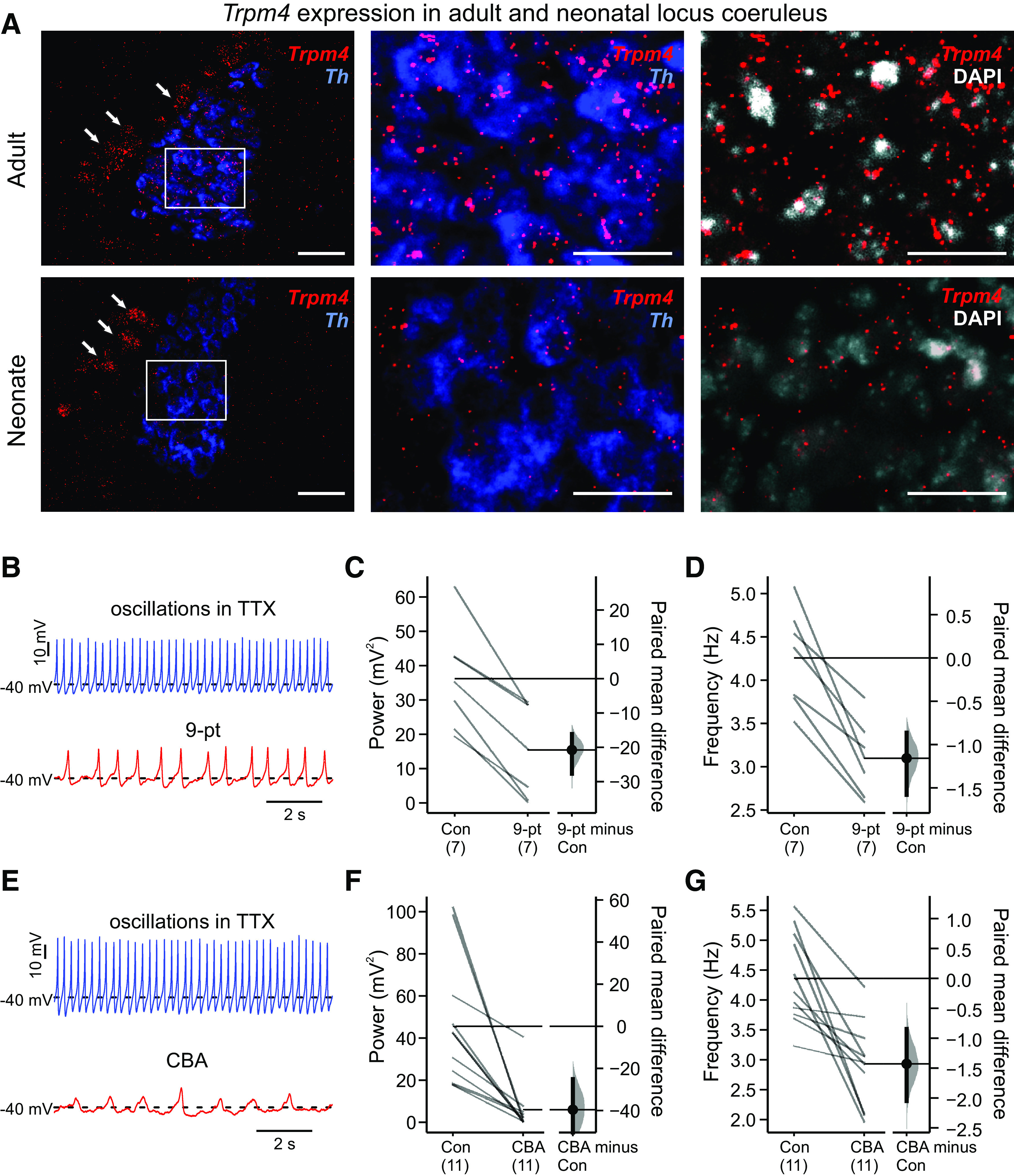
TRPM4 is expressed and contributes to subthreshold oscillations in LC neurons. ***A***, RNAscope analysis of *Th* (blue) and *Trpm4* (red) transcript expression in mouse LC neurons from adult (upper) and neonate (lower). The boxed regions in lower power images are shown at higher power (scale bars: 50 μm). Arrows point to laterally located MeV neurons also expressing *Trpm4*. ***B–G***, Exemplar effects on subthreshold oscillations in TTX (0.5 μm TTX, at −40 mV with DC current injection) of bath application of two TRPM4 channel blockers, 9-pt (***B***, 30 μm) and CBA (***E***, 50 μm), with summary for all cells showing paired mean differences as bootstrap sampling distributions for effects on integrated power (***C***, ***F***) and mean frequency (***D***, ***G***) after either 9-pt (***C***, ***D***) or CBA (***F***, ***G***); *p* < 0.0001 for all paired mean differences by two-sided permutation *t* test.

Expression of *Trpm4* (and *Trpm5* to a lesser extent) has been identified by qRT-PCR from microdissections of the LC region (Cui et al., 2011), and in a microarray analysis, *Trpm4* was found to be 17-fold enriched in LC neurons, relative to hindbrain (Mulvey et al., 2018). Therefore, we first performed multiplex fluorescence *in situ* hybridization to examine *Trpm4* expression specifically in catecholaminergic neurons of the LC, identified by co-expression of *Th*. We found *Trpm4* transcript labeling in *Th*-expressing LC neurons in sections from both neonatal and adult mice (Fig. 2*A*) at levels apparently similar to (adult) or slightly less (neonate) than observed in the mesencephalic trigeminal (MeV) cells located just laterally (Fig. 2*A*, arrows). Therefore, we used two chemically distinct TRPM4 channel blockers, 9-pt (30 μm) and CBA (50 μm), to test the contributions of TRPM4 to TTX-resistant subthreshold oscillations and their underlying currents in LC neurons (Grand et al., 2008; Guinamard et al., 2014; Burris et al., 2015; Ozhathil et al., 2018). At these concentrations, and consistent with previous results from RTN neurons (Li et al., 2021), we observed a strong reduction in both the power and frequency of the oscillations in LC neurons after exposure to either of the TRPM4 blockers (Fig. 2*B–G*). In both cases, a residual oscillation remained, perhaps reflecting incomplete TRPM4 block at the applied concentrations or contributions from other channels.

### Oscillations evoke calcium and TRPM4 currents in LC neurons

**Figure 3.**
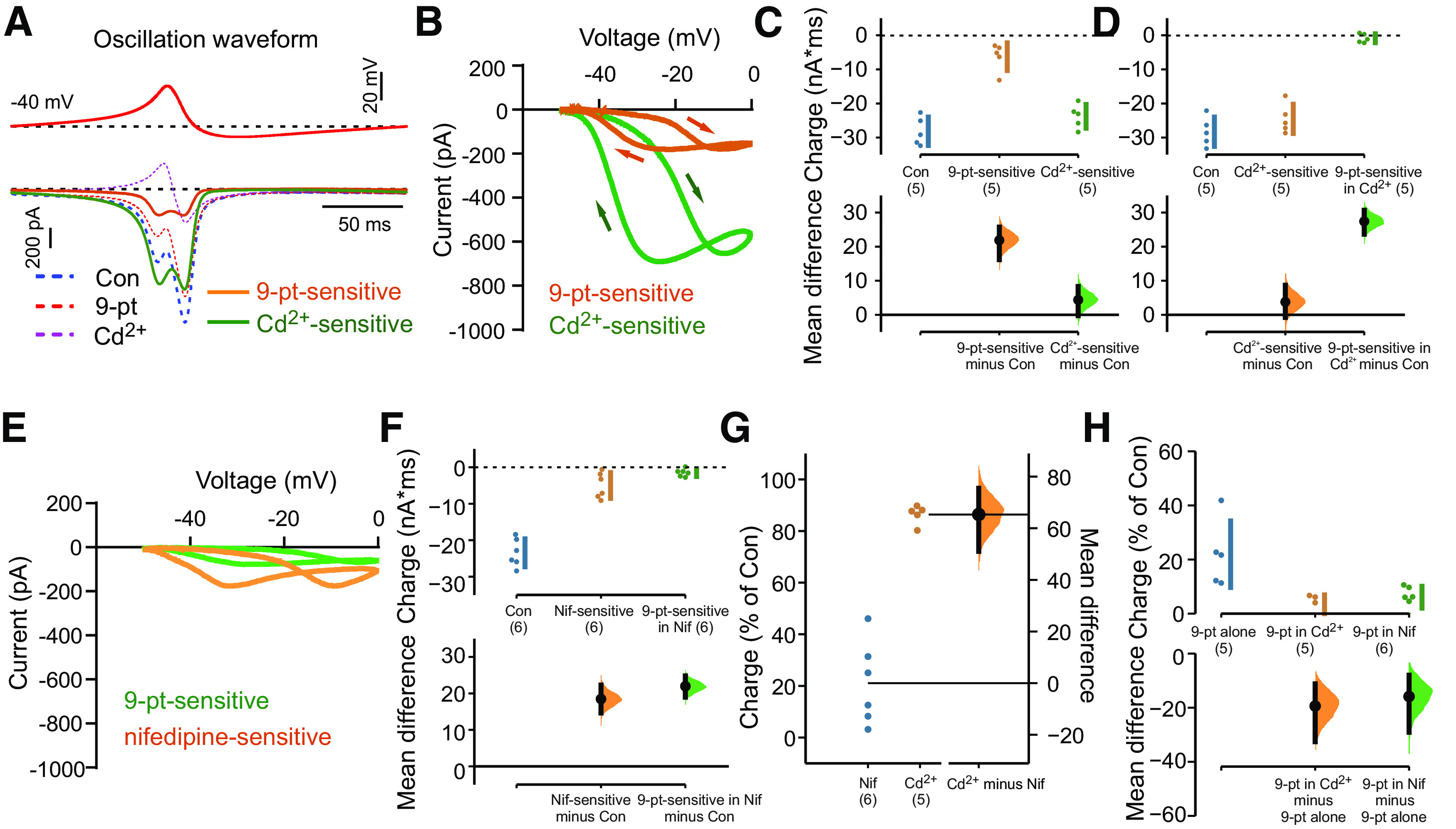
Calcium channels (L-type) and calcium-dependent TRPM4 currents contribute to subthreshold oscillations in LC neurons. ***A***, upper, Oscillation-waveform voltage command applied from Vhold = −40 mV in LC neurons recorded with a Cs^+^-based pipette solution. *Lower*, Oscillation-waveform-evoked currents under control conditions, in the presence of 9-pt (30 μm) and in the presence of 200 μm Cd^2+^ (dashed lines); 9-pt-sensitive and Cd^2+^-sensitive current components (thick lines) were obtained by digital subtraction. ***B***, I-V curves of oscillation-waveform-induced currents reveal inward 9-pt-sensitive and Cd^2+^-sensitive currents throughout the oscillation-waveform; in this experiment, 9-pt was applied before Cd^2+^. ***C***, ***D***, Summary data show the relative contributions of 9-pt-sensitive and Cd^2+^-sensitive currents during the oscillation waveform command (***C***) and illustrate that the 9-pt-sensitive current was eliminated when calcium currents were previously blocked with Cd^2+^ (***D***). The mean differences from control are presented on the lower plots as bootstrap sampling distributions. Data were assessed statistically by two-sided permutation *t* test (***C***, *p* = 0.0012 for 9-pt-sensitive and *p* = 0.114 for Cd^2+^-sensitive; ***D***, *p* = 0.195 for Cd^2+^-sensitive and *p* = 0.0012 for 9-pt-sensitive in Cd^2+^). ***E*,** Exemplar I-V curves of oscillation-waveform-induced currents during exposure to the L-type channel blocker nifedipine (10 μm) and then 9-pt (30 μm). ***F***, Summary data (*n* = 6) showing the relative contributions of nifedipine-sensitive and 9-pt-sensitive currents (after nifedipine) during the oscillation waveform command. The mean differences from control are presented on the lower plots as bootstrap sampling distributions. Data were assessed statistically by two-sided permutation *t* test (*p* < 0.0001 for nifedipine and for 9-pt in nifedipine). ***G***, Summary of the percentage of total charge during the waveform attributed to calcium current (Cd^2+^-sensitive) and L-type (nifedipine-sensitive) calcium channel current. The mean differences from Cd^2+^ are presented on the lower plots as bootstrap sampling distributions. For Cd^2+^-sensitive (*n* = 5) versus nifedipine-sensitive (*n* = 6): *p* < 0.0001, by two-sided permutation *t* test. ***H***, Summary of the percentage of total charge attributed to TRPM4 current under the indicated conditions. The mean differences from 9-pt-sensitive (*n* = 5): *p* = 0.006 for 9-pt-sensitive in Cd^2+^ (*n* = 5), and *p* = 0.003 for 9-pt-sensitive in nifedipine (*n* = 6) by two-sided permutation *t* test.

To examine contributions of calcium and TRPM4 channels activated during the membrane potential oscillation, we used whole-cell voltage clamp to measure ionic currents evoked by an oscillation waveform command derived from LC neurons (Fig. 3*A*, upper). Oscillation-waveform-evoked currents were recorded in the presence of TTX and fast synaptic blockers, and using a Cs^+^-based, TEA-containing pipette solution to eliminate K^+^ currents. The oscillation waveform elicited a biphasic inward current under control conditions, with an initial inward peak just before the waveform reached the maximal depolarization followed by a second, and larger, inward current peak during the repolarizing phase of the waveform (Fig. 3*A*, lower, dashed blue line). These oscillation waveform-evoked currents include passive leak and capacitive components, together with contributions from the ion channel currents of interest; the latter were isolated as the components of waveform-evoked current that are sensitive to various ion channel blockers.

We first exposed cells to 9-pt to test whether TRPM4 currents were activated during the oscillation voltage waveform. The 9-pt-sensitive current was revealed by digital subtraction of currents measured in the presence of 9-pt from those obtained under control conditions (Fig. 3*A*, lower, orange line). This 9-pt-sensitive, TRPM4-like current was also biphasic, with an initial inward current peak preceding the maximum depolarization and a second peak during the repolarizing phase of the waveform. When transformed against voltage, the 9-pt-sensitive current was inward throughout the oscillation waveform, with the two inward current peaks evident as a loop moving toward and away from the peak voltage in the current-voltage (I-V) plot (Fig. 3*B*, orange).

TRPM4 is a calcium-activated cationic channel, and previous observations in LC neurons have suggested a prominent role for voltage-activated calcium currents in generating the oscillations (Filosa and Putnam, 2003; Imber and Putnam, 2012; Sanchez-Padilla et al., 2014). Thus, to characterize the calcium current components evoked by the oscillation voltage waveform, we next tested effects of the nonspecific calcium channel blocker, Cd^2+^ (200 μm, in the continued presence of 9-pt). The Cd^2+^-sensitive current was much larger in amplitude than the 9-pt-sensitive current, although similarly biphasic, with two distinct peaks coinciding in both voltage and time with those of the 9-pt-sensitive current (Fig. 3*A–C*, green); these properties were also reflected in the I-V plot for the Cd^2+^-sensitive current, that was again inward throughout the waveform (Fig. 3*B*). The two peaks of Cd^2+^-sensitive current presumably result from activation/inactivation properties of different calcium channels with distinct voltage-dependence and kinetics, as those play out over the continually changing driving force for Ca^2+^ throughout the voltage waveform (i.e., decreasing during the depolarizing phase and then increasing again during membrane repolarization). The biphasic nature of the Ca^2+^-dependent TRPM4 cationic current (i.e., 9-pt-sensitive current) likely reflects similar changes in sodium driving force throughout the waveform, while also being influenced by the kinetics of the Ca^2+^ current evoked by the waveform. Consistent with the expected Ca^2+^-dependence of TRPM4, when Cd^2+^ was applied to block Ca^2+^ current before 9-pt exposure, the oscillation no longer evoked any 9-pt-sensitive current (Fig. 3*D*). This analysis indicates that Ca^2+^ channels and Ca^2+^-dependent TRPM4 channels contribute to the membrane potential oscillation in LC neurons, with Ca^2+^ channels accounting for the predominant current component.

In previous work from LC neurons, the subthreshold membrane potential oscillations were strongly inhibited by dihydropyridine L-type calcium channel antagonists (Filosa and Putnam, 2003; Imber and Putnam, 2012; Sanchez-Padilla et al., 2014). Therefore, we tested whether a nifedipine-sensitive, L-type calcium current was evoked by the oscillation waveform in LC neurons. The nifedipine-sensitive waveform-evoked current displayed I-V characteristics similar to the total waveform-evoked Ca^2+^ current (Fig. 3*E*,*F*), but it was much smaller in amplitude than the overall Cd^2+^-sensitive current (compare with Fig. 3*D*). Likewise, the 9-pt-sensitive current was still present but reduced in amplitude after blocking L-type channels with nifedipine (Fig. 3*E*,*F*). Overall, Cd^2+^-sensitive current carried the largest fraction of charge during the oscillation waveform (∼80%), including ∼20% that was dependent on L-type calcium channels (Fig. 3*G*). Approximately 20% of the inward charge could be ascribed to 9-pt-sensitive, TRPM4 channel current and, interestingly, although L-type channels accounted for only ∼25% of the total charge because of Ca^2+^ current, blocking those channels with nifedipine reduced the 9-pt-sensitive current by nearly as much as Cd^2+^ (i.e., by ∼70%; Fig. 3*H*). This suggests a preferential, although not exclusive, coupling between L-type Ca^2+^ channels and Ca^2+^-dependent TRPM4 channels.

### TRPM4 regulates spontaneous cell firing in LC neurons

**Figure 4.**
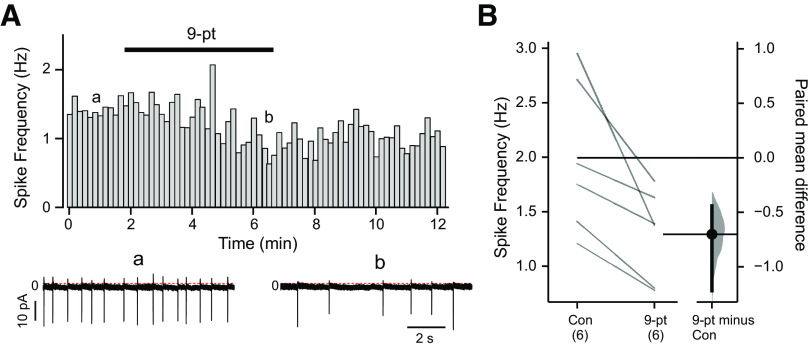
TRPM4 contributes to spontaneous firing in LC neurons. ***A***, Rate histogram (10-s bin averages of instantaneous frequency) of spontaneous action potential firing (no TTX) in an LC neuron recorded in cell-attached configuration, under control conditions and in the presence of 9-pt (30 μm). Example recordings from the indicated time points are provided (below). ***B***, Summary data show firing frequency for cells in control and 9-pt, and the paired mean difference between control and 9-pt as a bootstrap sampling distribution; *p* < 0.0001 by two-sided permutation *t* test.

To test whether TRPM4 plays a role in spontaneous pacemaker-like activity in LC neurons, we determined effects of 9-pt on action potential discharge using cell-attached recordings; this patch clamp configuration avoids disrupting the internal milieu of the recorded neuron. As illustrated for the exemplar LC neuron in Figure 4*A*, the steady metronomic-like firing typically observed under control conditions was reduced in frequency after bath application of 9-pt (30 uM). This 9-pt-induced decrease in firing frequency was consistently noted across all cells tested (Fig. 4*B*), indicating that TRPM4 participates in determining LC firing frequency. As we observed for its effects on subthreshold oscillations (Fig. 2*B–D*), spontaneous firing was not eliminated by 9-pt at this concentration.

### TRPM4 is expressed at low levels in SCN neurons

Subthreshold voltage-dependent membrane potential oscillations have been recorded in circadian-associated SCN neurons during the daytime, a period when SCN neurons are depolarized and fire spontaneously at higher frequencies than during the night (Pennartz et al., 2002; Jackson et al., 2004). A complex set of channels has been implicated in mediating the oscillations and cell firing, but a role for TRPM4 has not been tested (Pennartz et al., 2002; Jackson et al., 2004; Flourakis et al., 2015).

**Figure 5.**
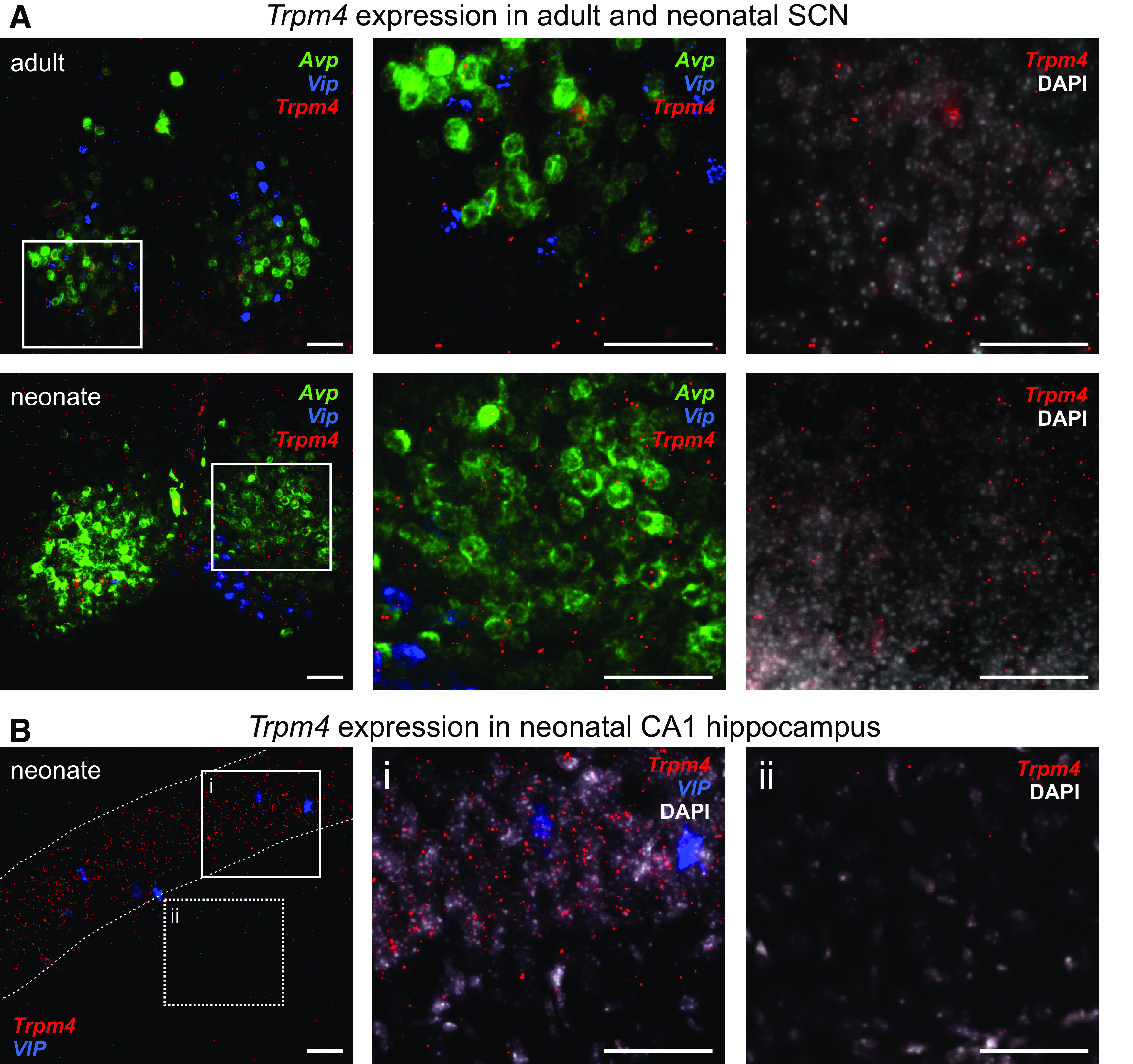
Trpm4 expression in SCN adult and neonatal SCN neurons. ***A***, RNAscope analysis of *Avp* (green), *Vip* (blue), and *Trpm4* (red) transcript expression in mouse SCN neurons from adult (upper) and neonate (lower). The boxed regions in lower power images are shown at higher power (and with a DAPI counterstain, right). ***B***, RNAscope analysis of *Vip* (blue) and *Trpm4* (red) in neonatal mouse hippocampus, with magnified images from the boxed regions shown with a DAPI counterstain to illustrate moderate *Trpm4* transcript levels (***Bi***; CA1) and background levels of labeling (***Bii***; SR, stratum radiatum). Dashed lines outline the CA1 region in ***B***; scale bars: 50 μm.

We first used multiplex RNAscope to assess *Trpm4* transcript expression in SCN neurons from adult and neonatal mice. The SCN is a heterogenous nucleus with at least five molecularly defined neuronal cell groups, each of which shows some degree of circadian rhythmicity (Wen et al., 2020). Among these, three distinct subsets of SCN neurons express either (or both) of the neuropeptides, *vasoactive intestinal polypeptide* (*Vip*) or *arginine vasopressin* (*Avp*), which are associated, respectively, with the core and shell regions of the SCN (Card et al., 1981; Abrahamson and Moore, 2001; Todd et al., 2020; Wen et al., 2020). Thus, to aid in SCN localization for these anatomic studies, we combined *Trpm4* localization with labeling for *Vip* and *Avp*. A low level of *Trpm4* expression was detectable in the SCN of both adult (Fig. 5A, upper) and neonate mice (Fig. 5*A*, lower) that was not obviously restricted to either the *Vip*^+^ and *Avp*^+^ neurons. As a reference control, note that the relative levels of *Trpm4* expression in SCN were roughly comparable to those in CA1 pyramidal cell region (Fig. 5*Bi*), and higher than the apparently background levels in stratum radiatum (Fig. 5*Bii*). These data suggest that TRPM4 channels are expressed in SCN neurons where they could contribute to subthreshold oscillations.

### TRPM4 in SCN neurons contributes to subthreshold oscillations

We next tested whether TRPM4 channels contribute to the subthreshold voltage-dependent membrane potential oscillations observed previously in SCN neurons (Pennartz et al., 2002; Jackson et al., 2004). We prepared brain slices from the mouse ventral forebrain and recorded from visually-identified neurons in SCN, recognizable as a cell-dense region at the base of the third ventricle immediately superior to the optic chiasm (from 0.82 to 0.34 mm caudal to bregma; Paxinos and Franklin, 2001). Recordings were not limited to the dorsomedial (shell) regions of the SCN, where oscillations were previously observed (Pennartz et al., 2002; Jackson et al., 2004).

**Figure 6.**
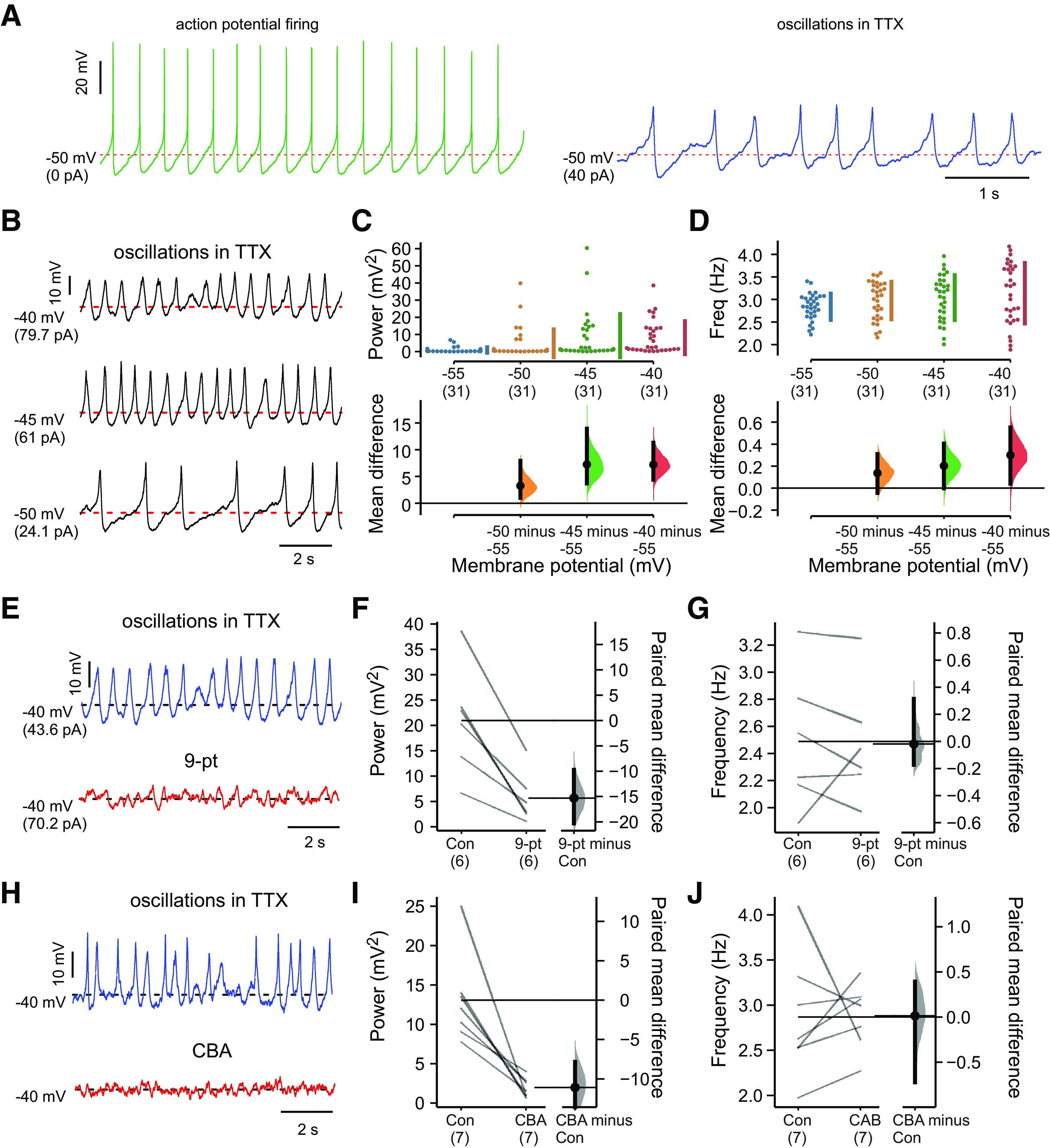
TRPM4 contributes to subthreshold oscillations in SCN neurons. ***A***, Action potentials and subthreshold oscillations recorded in the same neuron at −50 mV (by DC current injection). ***B***, Subthreshold oscillations at different membrane potentials in SCN neurons (in TTX, 0.5 μm). ***C***, ***D***, Integrated oscillation power (***C***) and frequency (***D***) obtained by FFT for multiple SCN neurons at different membrane potentials; oscillations were clearly observed in ∼77% of SCN neurons at −40 to −45 mV (*n* = 24/31, with integrated power >1). The mean differences from −55 mV are presented on the lower plots as bootstrap sampling distributions. Data were assessed statistically by two-sided permutation *t* test. For power: *p* = 0.0306 at −50 mV, *p* = 0.0004 at −45 mV, *p* < 0.0001 at −40 mV. For frequency: *p* = 0.14 at −50 mV, *p* = 0.0554 at −45 mV, *p* = 0.023 at −40 mV. ***E–J***, Representative effects on subthreshold oscillations in TTX (0.5 μm TTX, at −40 mV with DC current injection) of bath application of two TRPM4 channel blockers, 9-pt (***E***, 30 μm) and CBA (***H***, 50 μm), with summary for all cells showing paired mean differences as bootstrap sampling distributions for effects on integrated power (***F***, ***I***) and mean frequency (***G***, ***J***) after either 9-pt (***F***, ***G***) or CBA (***I***, ***J***). Paired mean differences for power: *p* < 0.0001 for 9-pt and *p* = 0.0122 for CBA; and for frequency: *p* = 0.904 for 9-pt and *p* = 0.967 for CBA, all by two-sided permutation *t* test.

In the presence of synaptic blockers, SCN neurons fired spontaneous action potentials that were blocked with TTX, often revealing a slower, large amplitude subthreshold oscillation (Fig. 6*A*; Pennartz et al., 2002; Jackson et al., 2004). In the presence of TTX, these subthreshold membrane potential oscillations were uncovered or enhanced in a substantial fraction of cells as they were depolarized with DC current injection (Fig. 6*B*). The membrane potential oscillations in SCN neurons were analyzed by FFT; in those neurons where they were evident (*n* = 24/31, ∼77%), the subthreshold oscillations were voltage-dependent, increasing in power and frequency at depolarized membrane potentials up to ∼−45 mV, with no further increase at −40 mV (Fig. 6*C*,*D*). We tested whether TRPM4 contributes to these oscillations in SCN neurons using 9-pt and CBA. Indeed, both TRPM4 blockers nearly eliminated the oscillations (Fig. 6*E*,*H*); interestingly, although the oscillation power was reduced by the blockers (Fig. 6*F*,*I*), the remaining membrane potential fluctuations continued at the same frequency as the larger oscillations (Fig. 6*G*,*J*).

### TRPM4 influences spontaneous cell firing in SCN neurons

**Figure 7.**
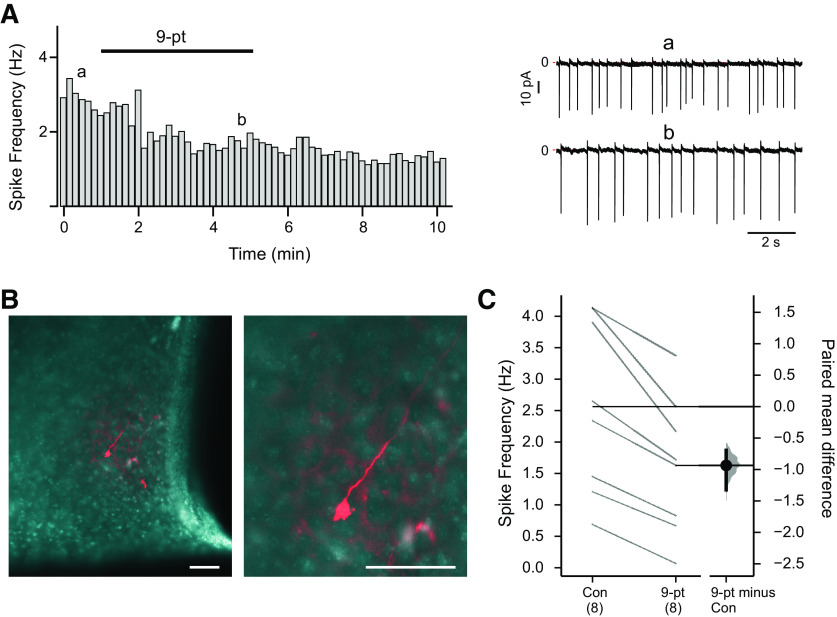
TRPM4 contributes to spontaneous firing in SCN neurons. ***A***, Rate histogram (10-s bin averages of instantaneous frequency) of spontaneous action potential firing (no TTX) in an SCN neuron recorded in cell-attached configuration, under control conditions and in the presence of 9-pt (30 μm). Example recordings from the indicated time points are provided (right). ***B***, Photomicrograph of Lucifer yellow-filled SCN neuron. ***C***, Summary data show firing frequency for cells in control and 9-pt and the paired mean difference between control and 9-pt as a bootstrap sampling distribution; *p* = 0.0096 by two-sided permutation *t* test. Scale bars: 50 μm.

Finally, we examined whether TRPM4 contributes to spontaneous action potential firing in SCN neurons. As depicted in Figure 7*A*, by cell-attached recording, we found that 9-pt decreased firing rate in SCN neurons; after whole-cell access with a Lucifer yellow-filled pipette and *post hoc* staining, we confirmed the cell locations in the SCN (Fig. 7*B*). This response was typical of all SCN neurons tested, in which the TRPM4 blocker consistently reduced baseline firing frequency without eliminating spontaneous firing (Fig. 7*C*). Thus, TRPM4 contributes to setting basal discharge rate in SCN neurons.

## Discussion

Subthreshold membrane potential oscillations are an uncommon, but important electroresponsive property that support spontaneous pacemaker-like action potential discharge in select neuronal populations. In earlier work, voltage-dependent TTX-resistant subthreshold oscillations were observed in LC and SCN neurons and, based on their sensitivity to dihydropyridine antagonists, L-type calcium channels were associated with those oscillatory events (Pennartz et al., 2002; Filosa and Putnam, 2003; Jackson et al., 2004; Imber and Putnam, 2012; Sanchez-Padilla et al., 2014). The current work extends that idea by demonstrating the additional involvement of TRPM4, a calcium-activated and voltage-dependent cationic channel in the oscillations of both LC and SCN neurons. This recalls the recent description of an overtly similar subthreshold membrane potential oscillation in mouse RTN neurons that was also dependent on L-type calcium channels and TRPM4 (Li et al., 2021). Thus, it appears that subthreshold oscillations require calcium entry, along with a depolarizing current, to activate TRPM4 channels and drive the oscillations in RTN, LC, and SCN neurons. In these neurons, we found that pharmacological inhibition of TRPM4 reduced, but did not eliminate, baseline action potential discharge rate (this work, and Li et al., 2021). Thus, although TRPM4 activity is necessary for the large amplitude TTX-resistant membrane potential oscillations and modulates firing rate in these neurons, it is not absolutely required for spontaneous pacemaker-like firing.

### TRPM4 and other channels regulate neuronal oscillations and firing

The subthreshold membrane potential properties that underlie spontaneous firing activity in LC and SCN neurons are complex, and involve factors other than the L-type calcium channels and TRPM4-dependent large amplitude oscillations that we examined here. For example, we find that L-type channels account for a relatively small fraction of the total Cd^2+^-sensitive calcium currents evoked by the waveform in LC neurons, even as they are responsible for nearly all of 9-pt-sensitive current and have been shown to strongly inhibit oscillations (Filosa and Putnam, 2003; Imber and Putnam, 2012; Sanchez-Padilla et al., 2014). Although low threshold L-type channels play an important role in generating subthreshold oscillations, dihydropyridine L-type channel blockers had only modest or variable effects on baseline action potential firing frequency in LC and SCN neurons (Filosa and Putnam, 2003; Jackson et al., 2004). In SCN neurons, an indirect consequence of L-type channel blockers was reduction of Ca^2+^-activated K^+^ current, which provided a depolarizing effect to substitute for the loss of calcium current and mitigate effects on firing (Jackson et al., 2004). The modest effects of L-type channel blockers on firing contrast with the relatively consistent and clear effect of TRPM4 blockers on firing frequency that we found here in LC and SCN neurons, and reported earlier in RTN neurons (Li et al., 2021). Interestingly, an important role for NALCN, a “leak” sodium channel, in driving spontaneous firing has also been reported in all three cell groups: LC, SCN, and RTN (Jackson et al., 2004; Sanchez-Padilla et al., 2014; Flourakis et al., 2015; Shi et al., 2016). Thus, we suspect that the large subthreshold oscillations and spontaneous firing in these cells are driven by an interplay between Ca^2+^ influx carried via low-threshold L-type calcium channels and depolarization mediated by NALCN channels, as integrated and amplified by the combined Ca^2+^-sensitive and voltage-sensitive TRPM4 cationic channels.

It is also important to note that earlier work characterized additional smaller subthreshold membrane potential fluctuations under both control and TTX conditions at more negative membrane potentials in rat LC neurons; we occasionally observed these small amplitude oscillations but did not examine them in detail. It was suggested that the smaller oscillations may be low amplitude manifestations of the larger “TTX-insensitive spikes” that they also observed at more depolarized potentials (e.g., since both are sensitive to L-type channel blockers; Christie et al., 1989; Filosa and Putnam, 2003; Imber and Putnam, 2012). The smaller oscillations may have been more prevalent in earlier work because of the absence of synaptic blockade and/or recordings performed at more physiological temperatures in a CO_2_-HCO_3_^–^-based bath solution with elevated (5 mm) K^+^ (Christie et al., 1989; Filosa and Putnam, 2003; Imber and Putnam, 2012).

We found subtle differences in how TRPM4 contributes to electroresponsive properties in LC and SCN neurons, perhaps reflecting the complement of supporting ion channels in the different cell groups. For example, TRPM4 blockers reduced the oscillation power in LC and SCN neurons but they lowered the frequency of the residual membrane potential fluctuations only in LC neurons. This suggests a particular role in LC neurons for TRPM4 in the oscillatory mechanism, per se, in addition to its more general amplifying effect on oscillatory power that was observed in the other cell groups. It is also possible that variable contributions from the related Ca^2+^-activated TRPM5 cation channel (Prawitt et al., 2003), for which there is currently no specific blocker, could account for some of these neuron-specific differences.

### TRPM4 and oscillations in other neurons

A role for TRPM4 is also possible in other cells where similar oscillations been recorded. For example, SNpc neurons express TRPM4 and the oscillation-dependent tonic firing of SNpc neurons is blocked by 9-pt and flufenamic acid, which also inhibits TRPM4 (Mrejeru et al., 2011). Likewise, TRPM4 mediates a plateau potential and persistent firing in reticular thalamic neurons following burst firing or membrane depolarization that contributes to the slow, 1-Hz, oscillations observed in thalamocortical circuits (O’Malley et al., 2020). However, the oscillatory phenomenon (network vs intrinsic), the source for calcium to enable TRPM4 activity (T-type vs L-type) and the ensuing cellular membrane potential property (plateau potential vs subthreshold oscillation) are all very different in this context. Interestingly, we also found that *Trpm4* is prominently expressed in MeV neurons, immediately adjacent to LC, and those cells also exhibit prominent subthreshold resonance and oscillations (Wu et al., 2001; Tanaka et al., 2003; Tsuruyama et al., 2013). It should be noted, however, that the characteristics of those MeV membrane phenomena are different from the oscillations in RTN, LC and SCN neurons, i.e., MeV neurons display ∼10-Hz oscillations at hyperpolarized potentials that require HCN current and ∼90-Hz oscillations at depolarized potentials that require TTX-sensitive persistent Na_V_ current, but not calcium current (Wu et al., 2001; Tanaka et al., 2003; Tsuruyama et al., 2013). Nonetheless, these distinct properties do not preclude a supporting role for TRPM4 current in MeV oscillations, a possibility that has not been tested.

### Limitations and caveats

Some limitations should be acknowledged. First, the experiments were performed on neurons in slices, where voltage-clamp conditions are not ideal; this can be especially of concern in cells with extensive processes or electrotonic coupling, as has been described for neonatal/adult LC and SCN neurons (Christie et al., 1989; Jiang et al., 1997). That said, such “space clamp” concerns are less acute when analyzing electroresponsive properties with relatively slow kinetics, as is the case with the oscillations we examined. Second, we used two channel blockers, 9-pt and CBA, to identify TRPM4 contributions to subthreshold oscillations in LC and SCN neurons. Although both blockers are reported to be relatively specific for the channel at the concentrations used (30 μm and 50 μm; Grand et al., 2008; Guinamard et al., 2014; Burris et al., 2015; Ozhathil et al., 2018), we cannot exclude off-target actions of these compounds. Indeed, in vascular cells, 9-pt was reported to activate a calcium-activated K^+^ current (Garland et al., 2015) and inhibit TMEM16A (Burris et al., 2015). In TRPM4 knock-out mice, 9-pt blocked a muscarinic-dependent sADP in pyramidal neurons of prefrontal cortex, implying an off-target action at the 100 μm concentration applied (Lei et al., 2014); by contrast, the effects of 9-pt on cardiac pacing were eliminated in TRPM4 knock-out mice, as expected for selective actions on the channel in this context (Guinamard et al., 2015). We are not aware of any reported nonspecific actions of the newer compound, CBA, in either cell systems or knock-out mice. Thus, these concerns are mitigated by the essentially identical actions obtained with two chemically distinct pharmacological blockers of the channel. Finally, we did not examine these oscillatory phenomena in TRPM4 knock-out mice, and we are also unaware of any reports of phenotypes in TRPM4 knock-out mice that can be attributed to altered function in LC and SCN neurons; some may yet be discovered.

In conclusion, together with recent work from RTN respiratory chemoreceptor neurons (Li et al., 2021), the current results from LC and SCN neurons support a general role for TRPM4 channels in providing a calcium-dependent and voltage-dependent cationic current to help drive the large subthreshold oscillations that support spontaneous firing in these cells.

## Synthesis

Reviewing Editor: Kevin Wickman, University of Minnesota Decisions are customarily a result of the Reviewing Editor and the peer reviewers coming together and discussing their recommendations until a consensus is reached. When revisions are invited, a fact-based synthesis statement explaining their decision and outlining what is needed to prepare a revision will be listed below. The following reviewer(s) agreed to reveal their identity: Michael Beierlein, Alexander Jackson.

In this manuscript, the authors investigate the contribution of TRPM4 conductance to subthreshold low-frequency membrane oscillations in noradrenergic neurons in the locus coeruleus (LC) and neurons of the suprachiasmatic nucleus (SCN). Prior to this study, these oscillations were thought to be mediated primarily by L-type Ca2+ channels, but recent work (Li et al, 2021) in neurons of the RTN - which display similar oscillations - revealed a contribution of Ca2+-dependent TRPM4 conductance. In this study, the authors build on this work, repeating several of the key experiments conducted in RTN neurons. The authors employed RNAscope to demonstrate TRMP4 expression in both LC and SCN neurons, and used slice electrophysiological approaches to show that TTX-resistant, voltage-dependent oscillations are attenuated by two different TRPM4 antagonists. Interestingly, blocking L-type Ca2+ channels in LC neurons strongly reduced the TRPM4-mediated current, suggesting that influx of Ca2+ via L-type Ca2+ channels is a critical regulator of TRPM4 activity in these neurons.

While not as exhaustive as the published study in RTN neurons, the present manuscript makes an important contribution by showing that TRPM4 conductances are more widespread than previously appreciated and could play prominent roles in shaping subthreshold oscillatory activity in various neuron populations. The data are of high quality and are clearly presented and analyzed. However, the manuscript would benefit substantially from a more thorough description of the data shown, clarification of some methods and experimental procedures, discussion of procedural limitations and confounds, and a more nuanced/balanced text (Abstract, Introduction, Results, and Discussion) that better integrates the published literature and examines alternative data interpretations. While additional electrophysiological experiments were identified (and presented below) that would strengthen the study, and the authors are encouraged to consider adding these data, the reviewers felt that these additional experiments are not essential. Attention to the TRPM4/RNAscope expression study in Figure 5 is essential, however, and this will likely require additional data.

Specific comments related to data presentation and related text content are as follows:

1) Related to the experiments presented in Figure 1, the authors should consider the following suggestions for manuscript improvement:

a. It would be useful to point to previous descriptions of this phenomenon as described in papers such as Filosa and Putnam, 2003 or Sanchez-Padilla et al, 2014. It is not clear, for example, whether bath application of TTX eliminates spontaneous pacemaking and results in the cell sitting at -50mV without any subthreshold oscillation or if hyperpolarizing DC current has to be applied to allow the cell to sit at -50mV.

b. Are subthreshold oscillations only evident when depolarizing DC current is injected? In other descriptions of this phenomenon, application of TTX often results in the spontaneous appearance of a stable subthreshold oscillation. A clarification of this point in the Results or Discussion would be helpful.

c. In the 2003 study from Filosa and Putnam, the authors differentiated between spontaneous subthreshold oscillations measured in the absence of TTX (events that resembled spikelets occurring during/in-between natural pacemaking) and the larger rhythmic subthreshold oscillations occurring at more depolarized potentials seen in the presence of TTX. It would be useful to comment on this distinction as well in the Discussion.

d. A brief description of methodological differences between the current and previous studies (e.g., rat vs. mouse, internal solutions, recording temperature, recordings made with or without synaptic blockers, etc.) that may account for differences in the descriptions of this phenomenon would be helpful.

2) Related to the experiments presented in Figure 2, the authors should consider the following suggestions for manuscript improvement:

a. A discussion of the literature supporting the selectivity of both 9-pt and CBA is important (beyond the reference to Li et al, 2021). This is a key point given that the authors do not manipulate TRPM4 expression (knockout or knock-down) and instead rely entirely on the selectivity of these drugs to frame their conclusions about TRPM4 relevance. How selective are these compounds for TRPM4 and are there off-target effects that could explain their effect on excitability? Have these compounds been used in studies using TRPM4 knockout mice, and are 9-pt- or CBA-sensitive currents absent in this mouse?

b. It would be helpful to demonstrate whether or not spontaneous firing in the absence of TTX is affected by 9-pt or CBA. This would appear to be an important determinant of whether TRPM4 is affecting natural pacemaking and not just oscillations in TTX.

c. Particularly relevant to the previous literature concerning the role of TRPM4 in oscillatory firing behavior, and the use of TRPM4 pharmacological tools in slices, is a 2020 study by O’Malley and colleagues in J Neurosci. Notably, this work is not cited in this manuscript. Comparison of the authors’ data with this published work should be a part of the Results or Discussion.

d. Relevant to the in situ hybridization data, it is interesting that MesV neurons appear to express TRPM4 at high levels. Notably, these neurons have also been shown to exhibit subthreshold oscillations and membrane resonance - primarily work from Scott Chandler (e.g., Wu et al, 2001). This could be an additional discussion point if the authors chose, as a way of further correlating TRPM4 with cell types that are capable of rhythmic subthreshold oscillations.

3) Related to the experiments presented in Figure 3, the authors should consider the following suggestions for manuscript improvement:

a. There appears to be a big discrepancy in the effect of blocking TRPM4 in voltage and current clamp. For example, while 9-pt and CBA have a strong effect on oscillations in current clamp (Fig. 2B-E), the actual charge carried by the 9-phen sensitive current recorded in voltage clamp is more modest (Fig. 3F). There are probably reasonable explanations for this difference. For example, depolarizations mediated by TRPM4 activation might in turn activate HVA currents, so even though the current carried by TRPM4 is small, it is critical for proper oscillations. The authors should better explain these results.

b. It is not clear if the nifedipine-sensitive component (Fig. 3D-F) is recorded in the presence of 9-pt or not; this point should be clarified. Regardless, the data show that L-type Ca2+ channels play a modest role during oscillations as compared to the remaining voltage-gated Ca2+ channels, which are not further identified here. However, the manuscript makes it seem that L-type Ca2+ channels are the main contributor to inward currents during TTX-insensitive oscillations. For example, the Abstract notes “...providing an ICaN to augment the L-type calcium current previously associated with this distinctive electro-responsive property.”, which is in direct contradiction to the data shown here. The authors should revise the text, so that the suggested role of L-type Ca2+ channels is better aligned with their data.

c. Although the oscillation waveform is slow, space-clamp is still a confound with this approach and the authors should state the limitations of this experiment and caveats in its interpretation.

d. It is unclear what the sequence of drug applications were in this experiment. Were 9-pt and Cd2+ applied independently and then together or were these separate experiments? Also, what is the rationale for using 9-pt as a TRPM4 blocker instead of CBA?

e. An additional way to strengthen the link between L-type channels and TRPM4 is by testing whether application of BayK8644 enhances the 9-pt-sensitive current; this is not essential, but rather a suggestion for potential future studies.

4) Related to the experiments presented in Figure 4, the authors should consider the following suggestions for manuscript improvement:

a)It would be helpful to represent the SCN data in a way that is consistent with the LC data (e.g., showing where in the SCN cells were targeted and some anatomical data). In addition, it would be useful to include a trace showing a comparison of spontaneous firing with the oscillations. Finally, showing the Power vs. Membrane Potential data in Fig. 4B in the same way as the authors showed it in Fig. 1F would be helpful.

b) The authors state that they recorded from “visually-identified SCN neurons”. However, the anatomical location within the SCN and the identify of SCN cell types is important to consider. The neurons that were shown to exhibit subthreshold oscillations in the presence of TTX in previous work (Pennartz et al, 2002; Jackson et al, 2004) were specifically from the dorsomedial or shell region of the SCN, which are enriched in AVP-expressing neurons and thought to be the site of autonomous circadian pacemaking. It is unclear whether VIP-expressing neurons in the core region of the SCN, which are the retino-recipient population, also exhibit the same subthreshold oscillations.

c) As was noted above for the LC, missing here is a demonstration of whether or not spontaneous firing in the absence of TTX is affected by 9-pt or CBA.

5) Related to the experiments presented in Figure 5, the authors need to address the following points - with additional experiments as needed - as part of the revision:

a. The RNAscope data for hippocampus (Fig. 5B-C) seem out of place and without a clear motivation; the authors are advised to remove these data from the manuscript.

b. As noted above, all work on subthreshold oscillatory activity in the SCN has been done on dmSCN neurons, which express AVP. This makes it puzzling why VIP is used as a cell type maker in the RNAScope experiment in Fig. 5A.

c. Also in Fig. 5A, there are little to no anatomical markers that define the boundaries of the SCN and importantly, and in contrast to the convincing results presented in Fig. 2A, the RNAScope experiments here do not convincingly show that TRPM4 is expressed in VIP neurons of the SCN. The more relevant question is whether TRPM4 is expressed in AVP-expressing neurons of the dmSCN.

d. The authors can strengthen their claim that TRPM4 is expressed in the SCN by probing publically available single-cell RNAseq databases including a recent scRNAseq atlas of the SCN (Wen et al, 2020, Nature Neuroscience).

Additional concerns that should be addressed in the revised manuscript are as follows:

1) The authors focus most of their efforts on oscillations recorded in the presence of TTX, which does not fully address the issue of how TRPM4 contributes to cell excitability under physiological conditions. The assumption here is that the subthreshold oscillations seen in the presence of TTX are the subthreshold drivers of pacemaking under unperturbed conditions. But an alternative view is that the oscillations measured in the presence of TTX are in fact a meta-phenomenon unique to conditions under which voltage-gated Na+ channels are blocked, and are most apparent in more depolarized voltage ranges. In principle, if subthreshold oscillations drive pacemaking, and TRPM4 channels drive subthreshold oscillations, then if follows that TRPM4 blockers should diminish pacemaking. Additionally, previous work carried out in SCN neurons (Jackson et al, J Neurosci 2004) suggests that L-type channel activation during normal action potential firing is modest, which would in turn limit the contribution of TRPM4. While adding additional data addressing these issues would strengthen the manuscript considerably, the role of TRPM4 during normal spike firing should at least be discussed.

2) The way that the general problem of ionic mechanisms of pacemaking is framed in the Abstract, Introduction and Discussion could be more nuanced. For example, the authors focus on previous work in the SCN showing that L-type Ca2+ current provides the inward current driving oscillatory activity, and cite work from Pennartz/2002 and Jackson/2004. In fact, Pennartz and colleagues first implicated a low-threshold L-type current in providing the depolarizing drive at subthreshold voltages, during the subjective day. While Jackson and colleagues showed that the low-threshold L-type current is there, its primary role appeared to be driving activation of Ca2+-activated K+ channels, and that a Na+ leak current was the more important contributor to interspike depolarization. This latter current was later identified as likely carried by NALCN channels (Flourakis et al, 2015, Cell), which is not cited in this manuscript (and should be). In light of this, the Discussion could provide a more detailed comparison of the current work and previous work and tie together possible synergistic interactions between low-threshold L-type current, NALCN and TRPM4 in circadian pacemaking.

Minor comments

1) The authors should replace ICaN with ICAN in the Abstract, which is the usual abbreviation for Ca2+-activated nonselective cation current.

2) The experiments in this manuscript were conducted at room temperature, which might have strong effects on intracellular Ca2+ signaling and, consequently, TRPM4 activation. Was there a reason these recordings were not or could not be carried out at more physiological temperatures?

3) Another point of clarification is whether the authors accounted for liquid junction potential in their current-clamp recordings and in voltage-clamp/oscillation-clamp recordings; this is a particularly important point to clarify given that they used different internal solutions in these experiments.

4) Also missing is a discussion of the TRPM4 knockout mouse - particularly whether this model exhibits any behavioral or physiological defects attributable to LC or SCN function (e.g., Circadian deficits).

5) The individual data points in Fig 3C, E and F are very large relative to the box-whisker plots, such that the data points mostly obscure them. This should be adjusted for clarity.

## Author Response

Synthesis Statement for Author (Required):

In this manuscript, the authors investigate the contribution of TRPM4 conductance to subthreshold lowfrequency membrane oscillations in noradrenergic neurons in the locus coeruleus (LC) and neurons of the

suprachiasmatic nucleus (SCN). Prior to this study, these oscillations were thought to be mediated primarily by L-type Ca2+ channels, but recent work (Li et al, 2021) in neurons of the RTN - which display similar oscillations - revealed a contribution of Ca2+-dependent TRPM4 conductance. In this study, the authors build on this work, repeating several of the key experiments conducted in RTN neurons. The authors

employed RNAscope to demonstrate TRMP4 expression in both LC and SCN neurons, and used slice electrophysiological approaches to show that TTX-resistant, voltage-dependent oscillations are attenuated

by two different TRPM4 antagonists. Interestingly, blocking L-type Ca2+ channels in LC neurons strongly

reduced the TRPM4-mediated current, suggesting that influx of Ca2+ via L-type Ca2+ channels is a critical regulator of TRPM4 activity in these neurons.

While not as exhaustive as the published study in RTN neurons, the present manuscript makes an important contribution by showing that TRPM4 conductances are more widespread than previously appreciated and could play prominent roles in shaping subthreshold oscillatory activity in various neuron populations. The data are of high quality and are clearly presented and analyzed. However, the manuscript

would benefit substantially from a more thorough description of the data shown, clarification of some

methods and experimental procedures, discussion of procedural limitations and confounds, and a more

nuanced/balanced text (Abstract, Introduction, Results, and Discussion) that better integrates the published literature and examines alternative data interpretations. While additional electrophysiological experiments were identified (and presented below) that would strengthen the study, and the authors are

encouraged to consider adding these data, the reviewers felt that these additional experiments are not

essential. Attention to the TRPM4/RNAscope expression study in Figure 5 is essential, however, and this

will likely require additional data.

We thank the Reviewers for a positive review of the manuscript, and for constructive suggestions for

improvement. We also thank you for your patience as productivity in our mouse husbandry hit an unexplained and unfortunate lull, temporarily limiting availability of experimental subjects.

We have addressed the specific comments in detail below, and hope you agree that our responses and

revisions have further improved the paper. We note here that we chose to perform the recommended

electrophysiological experiments, even though they were considered non-essential. We think the addition of these data, which show that TRPM4 blockers reduce spontaneous firing frequency in both LC and

SCN neurons, provide important support for the idea that the channel helps set the tonic firing rate in

these cell groups. We also performed additional RNAscope experiments to reveal both the Avp- and Vipcontaining populations of SCN neurons (new Fig. 5), and to verify the low, but detectable, Trpm4 transcript expression levels in SCN. We also appreciated the suggestion to expound in more detail about

ionic mechanisms for pacemaking, which we have done at various points in the revised manuscript; we

have also provided a more substantial discussion section to place our results in better context.

Finally, we re-made all the figures to present the data using the estimation statistics approach (previously included in the source data file), as requested by the Senior Editor (email of June 8).

Specific comments related to data presentation and related text content are as follows:

1) Related to the experiments presented in Figure 1, the authors should consider the following suggestions for manuscript improvement:

eN-NWR-0212-21

a. It would be useful to point to previous descriptions of this phenomenon as described in papers such as

Filosa and Putnam, 2003 or Sanchez-Padilla et al, 2014. It is not clear, for example, whether bath application of TTX eliminates spontaneous pacemaking and results in the cell sitting at -50mV without any

subthreshold oscillation or if hyperpolarizing DC current has to be applied to allow the cell to sit at -

50mV.

Yes, like Filosa and Putnam (2003) who found the resting membrane potential of LC neurons ranged

from -45 mV to -60 mV, we find that tonic firing activity in LC neurons arises from membrane potentials

around -50 mV. In addition, as they did also, we often observed a slight membrane depolarization after

application of TTX to eliminate spontaneous action potentials that required some hyperpolarizing DC

current injection to return the cell back to -50 mV. An example of this is illustrated below, and this similarly accounts for the negative DC current (-11.6 pA) displayed on Fig. 1D. We did not study the mechanism for this TTX-evoked membrane depolarization.

b. Are subthreshold oscillations only evident when depolarizing DC current is injected? In other descriptions of this phenomenon, application of TTX often results in the spontaneous appearance of a stable

subthreshold oscillation. A clarification of this point in the Results or Discussion would be helpful.

We note that the large subthreshold oscillations we examined were observed when DC current was applied to depolarize the membrane potential beyond -50 mV (p. 5)

c. In the 2003 study from Filosa and Putnam, the authors differentiated between spontaneous subthreshold oscillations measured in the absence of TTX (events that resembled spikelets occurring during/in-between natural pacemaking) and the larger rhythmic subthreshold oscillations occurring at more depolarized potentials seen in the presence of TTX. It would be useful to comment on this distinction as well in

the Discussion.

We now note that the subthreshold oscillations we observed in the presence of TTX were analogous to

the “TTX-insensitive spikes” of Filosa and Putnam (2003), and the “spikelets” of Sanchez-Padilla (p. 5).

We also distinguish those larger amplitude oscillations we both observed at depolarized potentials

(“TTX-insensitive spikes”) from the smaller membrane potential oscillations Filosa and Putnam observed

at more negative membrane potential, and note that they suggested these both may reflect the same

underlying phenomenon (p. 11).

d. A brief description of methodological differences between the current and previous studies (e.g., rat

vs. mouse, internal solutions, recording temperature, recordings made with or without synaptic blockers,

etc.) that may account for differences in the descriptions of this phenomenon would be helpful.

eN-NWR-0212-21

Although we do not see major discrepancies between our work and that of Filosa and Putnam (2003),

we now point out that we did not examine the smaller oscillatory events that were occasionally observed at more negative membrane potentials (see figure above) and we list some of the methodological differences that may account for a greater prevalence of those events in the earlier work (p. 11).

2) Related to the experiments presented in Figure 2, the authors should consider the following suggestions for manuscript improvement:

a. A discussion of the literature supporting the selectivity of both 9-pt and CBA is important (beyond the

reference to Li et al, 2021). This is a key point given that the authors do not manipulate TRPM4 expression (knockout or knock-down) and instead rely entirely on the selectivity of these drugs to frame their

conclusions about TRPM4 relevance. How selective are these compounds for TRPM4 and are there offtarget effects that could explain their effect on excitability? Have these compounds been used in studies

using TRPM4 knockout mice, and are 9-pt- or CBA-sensitive currents absent in this mouse?

We regret the oversight in not providing the appropriate references at the point in the Results where we

first mention our use of 9-pt and CBA - this is now corrected (p. 6). In addition, in a new section of the

Discussion we address some caveats and limitations with this pharmacological approach, and note some

other known ion channel targets and effects of the compounds in knockout mice (p. 12-13).

b. It would be helpful to demonstrate whether or not spontaneous firing in the absence of TTX is affected

by 9-pt or CBA. This would appear to be an important determinant of whether TRPM4 is affecting natural

pacemaking and not just oscillations in TTX.

We thank the Reviewers for suggesting this experiment. We used cell-attached recordings to test effects

of 9-phenanthrol on spontaneous firing in LC neurons. Firing rate was reduced, but not blocked, in the

presence of 9-pt. This indicates that TRPM4 participates in determining LC firing frequency, but that it is

not required to maintain ongoing discharge. These data are presented in a new Fig. 4.

c. Particularly relevant to the previous literature concerning the role of TRPM4 in oscillatory firing behavior, and the use of TRPM4 pharmacological tools in slices, is a 2020 study by O’Malley and colleagues in J

Neurosci. Notably, this work is not cited in this manuscript. Comparison of the authors’ data with this

published work should be a part of the Results or Discussion.

The paper referenced here (O’Malley et al., J Neurosci, 2020) identified a role for T-type calcium channels and TRPM4 in mediating a plateau potential and persistent firing in reticular thalamic neurons following burst firing or membrane depolarization; this intrinsic mechanism was suggested to contribute to

the slow, 1 Hz, oscillations that are observed in thalamocortical circuits. The oscillatory phenomenon

(network vs. intrinsic), the source for calcium to enable TRPM4 activity (T-type vs. L-type) and the ensuing cellular membrane potential property (plateau potential vs. subthreshold oscillation) are all very different in these two contexts. We now provide a new section in the Discussion that describes this and

other possible TRPM4-mediated oscillatory phenomena (p. 12).

d. Relevant to the in situ hybridization data, it is interesting that MesV neurons appear to express TRPM4

at high levels. Notably, these neurons have also been shown to exhibit subthreshold oscillations and

membrane resonance - primarily work from Scott Chandler (e.g., Wu et al, 2001). This could be an additional discussion point if the authors chose, as a way of further correlating TRPM4 with cell types that

are capable of rhythmic subthreshold oscillations.

Thanks for this suggestion. We have now added a comment regarding the prominent expression of

TRPM4 in MeV neurons, and the possibility that this channel could contribute to MeV subthreshold oscillations and membrane resonance. We note that these oscillatory phenomena in MeV neurons appear

distinctly different in key characteristics (e.g., 10 Hz and 90 Hz resonance frequencies at hyperpolarized

eN-NWR-0212-21

and depolarized membrane potentials, respectively) and critical ionic currents (Ih and TTX-sensitive INap) - and point out that this does not preclude some contribution from TRPM4 (p. 12).

3) Related to the experiments presented in Figure 3, the authors should consider the following suggestions for manuscript improvement:

a. There appears to be a big discrepancy in the effect of blocking TRPM4 in voltage and current clamp.

For example, while 9-pt and CBA have a strong effect on oscillations in current clamp (Fig. 2B-E), the actual charge carried by the 9-phen sensitive current recorded in voltage clamp is more modest (Fig. 3F).

There are probably reasonable explanations for this difference. For example, depolarizations mediated

by TRPM4 activation might in turn activate HVA currents, so even though the current carried by TRPM4 is

small, it is critical for proper oscillations. The authors should better explain these results.

We believe the Reviewer is correct - i.e., the size of the currents carried by the different channels under

an imposed oscillation waveform does not necessarily translate to their relative importance in initiating/supporting the natural oscillation. For example, the dihydropyridine-sensitive, low-threshold L-type

calcium channels are well-positioned to play a critical role in activating TRPM4 and initiating the oscillation (as has been demonstrated previously in these cells). The other calcium channel currents evoked

during the oscillation, as revealed by the evoked voltage clamp waveform, may not be necessary for initiating the oscillation.

b. It is not clear if the nifedipine-sensitive component (Fig. 3D-F) is recorded in the presence of 9-pt or

not; this point should be clarified. [break by authors]

We have clarified the order of presentation of the channel blockers for the different experiments, as

highlighted (p. 6-8). Here, we note for your convenience that for data in Fig. 3A-C, 9-pt application preceded Cd while in Fig. 3D, Cd application came first. For Fig. 3E,F, the order of application was first nifedipine and then 9-pt in nifedipine. For Fig. 3G the data for Cd and nifedipine are from experiments

where they were presented first; and, as noted on the figure panel for Fig. 3H, 9-pt data are from when

the TRPM4 blocker was presented first, or in the presence of either Cd or nifedipine.

Regardless, the data show that L-type Ca2+ channels play a modest role during oscillations as compared

to the remaining voltage-gated Ca2+ channels, which are not further identified here. However, the manuscript makes it seem that L-type Ca2+ channels are the main contributor to inward currents during TTXinsensitive oscillations. For example, the Abstract notes “...providing an ICaN to augment the L-type calcium current previously associated with this distinctive electro-responsive property.”, which is in direct

contradiction to the data shown here. The authors should revise the text, so that the suggested role of Ltype Ca2+ channels is better aligned with their data.

We think our results support a preferential (but not exclusive) role for L-type channels in activating

TRPM4 channels based on the disproportionately large effect of nifedipine on the 9-pt-sensitive current

evoked by the oscillation waveforms. That is, ∼70% of the TRPM4 current was reduced by nifedipine

even though L-type channels accounted for just 20% of the overall Cd-sensitive current (p. 8, 10-11).

In addition, we do not believe that our data are in discord with previous results suggesting a prominent

role for L-type channels in mediating the oscillations. As mentioned in the response above (point 3a),

low threshold L-type channels may be particularly important in triggering the oscillations even if they

ultimately contribute less overall calcium current throughout the entire waveform. Although we did not

re-examine this point here, the critical role for L-type channels in the oscillations was found in previous

work from LC and SCN neurons (and RTN and SNpc neurons). Based on these earlier observations, we

consider it likely that the dihydropyridine antagonists block this preferential L-type “trigger” calcium

eN-NWR-0212-21

source, and thereby interfere with initiation of the oscillation; absent the depolarization from the oscillation, the calcium current carried by other Ca channels in the imposed waveform would not occur. In

this way, a crucial role for L-type channels in triggering the oscillation is not inconsistent with greater Ca

current carried by other channels over the course of the waveform.

c. Although the oscillation waveform is slow, space-clamp is still a confound with this approach and the

authors should state the limitations of this experiment and caveats in its interpretation.

We now provide a Discussion section listing some limitations and caveats, including the issue of space

clamp in these slices (p. 12-13).

d. It is unclear what the sequence of drug applications were in this experiment. Were 9-pt and Cd2+ applied independently and then together or were these separate experiments? Also, what is the rationale

for using 9-pt as a TRPM4 blocker instead of CBA?

As mentioned above, we have provided a description of the order of drug application in the Results sections (p. 6-8).

We did not have any particular rationale for choosing 9-phenanthrol over CBA for these experiments;

both are potent and relatively selective for TRPM4 (see 2a, above). In all our assays to date these two

compounds have yielded essentially identical results (in these experiments and our earlier work on RTN

neurons). One (minor) point in favor of 9-pt is that it appears more often than CBA in the existing literature as a TRPM4 blocker.

e. An additional way to strengthen the link between L-type channels and TRPM4 is by testing whether

application of BayK8644 enhances the 9-pt-sensitive current; this is not essential, but rather a suggestion

for potential future studies.

This is an interesting idea. Because the role of L-type channels has previously been described, and our

focus here was to test a role specifically for TRPM4, we will take the Reviewer’s suggestion and leave

this for future studies.

4) Related to the experiments presented in Figure 4, the authors should consider the following suggestions for manuscript improvement:

a) It would be helpful to represent the SCN data in a way that is consistent with the LC data (e.g., showing where in the SCN cells were targeted and some anatomical data). In addition, it would be useful to

include a trace showing a comparison of spontaneous firing with the oscillations. Finally, showing the

Power vs. Membrane Potential data in Fig. 4B in the same way as the authors showed it in Fig. 1F would

be helpful.

We now present the SCN data to better match our presentation of the LC results (Fig. 6), including an

example of spontaneous firing and oscillations in the same cell. We also included an example of a Lucifer

Yellow-filled cell recorded from the SCN in Fig. 7B.

b) The authors state that they recorded from “visually-identified SCN neurons”. However, the anatomical

location within the SCN and the identify of SCN cell types is important to consider. The neurons that were

shown to exhibit subthreshold oscillations in the presence of TTX in previous work (Pennartz et al, 2002;

Jackson et al, 2004) were specifically from the dorsomedial or shell region of the SCN, which are enriched

in AVP-expressing neurons and thought to be the site of autonomous circadian pacemaking. It is unclear

whether VIP-expressing neurons in the core region of the SCN, which are the retino-recipient population,

also exhibit the same subthreshold oscillations.

eN-NWR-0212-21

We now indicate that recordings were not limited to the dorsomedial (shell) regions of the SCN, where

oscillations were previously observed (p. 9).

c) As was noted above for the LC, missing here is a demonstration of whether or not spontaneous firing

in the absence of TTX is affected by 9-pt or CBA.

As for the LC, we have also now tested effects of 9-phenanthrol on spontaneous firing in SCN neurons.

Consistent with a role for TRPM4, we found a consistent decrease in SCN firing rate in the presence of 9-

pt, although spontaneous discharge was not blocked. These data are presented in a new Fig. 7.

5) Related to the experiments presented in Figure 5, the authors need to address the following points -

with additional experiments as needed - as part of the revision:

a. The RNAscope data for hippocampus (Fig. 5B-C) seem out of place and without a clear motivation; the

authors are advised to remove these data from the manuscript.

We have chosen to retain RNAscope data from the hippocampus, although it is now less prominent. We

believe these data provide an important technical control given the relatively low levels of Trpm4 transcript expression in LC and SCN. The levels of Trpm4 expression presented across different hippocampal

regions provide examples of moderate (CA1) to negligible signal (stratum radiatum), allowing us to more

convincingly conclude that Trpm4 expression in LC and SCN is indeed above background. We now note

that these data are presented as a “reference control” for expression levels (p. 9)

b. As noted above, all work on subthreshold oscillatory activity in the SCN has been done on dmSCN neurons, which express AVP. This makes it puzzling why VIP is used as a cell type maker in the RNAScope experiment in Fig. 5A.

Thank you for this suggestion. We have now performed RNAscope histochemistry to simultaneously detect expression of Avp, Vip and Trpm4 transcripts in the SCN. These data are presented in a new Fig. 5.

c. Also in Fig. 5A, there are little to no anatomical markers that define the boundaries of the SCN and importantly, and in contrast to the convincing results presented in Fig. 2A, the RNAScope experiments here

do not convincingly show that TRPM4 is expressed in VIP neurons of the SCN. The more relevant question

is whether TRPM4 is expressed in AVP-expressing neurons of the dmSCN.

We believe the addition of Avp to the previous Vip labeling, in response to the Reviewer suggestion

above, has now provided a helpful demarcation of the outer shell region of the SCN. We note here that

Trpm4 expression in SCN is indeed associated with the Avp-expressing shell region, but our data do not

let us conclude that it is excluded from Vip-containing cells.

d. The authors can strengthen their claim that TRPM4 is expressed in the SCN by probing publically available single-cell RNAseq databases including a recent scRNAseq atlas of the SCN (Wen et al, 2020, Nature

Neuroscience).

Thank you for this suggestion. We accessed the data from Wen et al. 2020 for analysis by our Bioinformatics Core. However, the available data files did not include cluster annotations and did not permit access to cluster-level information without re-analyzing their data from the scratch. Nevertheless, as a first

approximation, we queried their database (across all CT points) selecting cells based on expression of

the pairs of marker genes identified for each of the 5 neuron clusters - and found that only a minor fraction detectably expressed Trpm4 (<5% each), and then only at low levels. Likewise, by that same analysis, those same cell groups express similarly low levels of Nalcn (albeit in a higher fraction of cells,

∼20%). In our view, the relatively modest sequencing depth available from the Drop-Seq method used

by Wen et al. 2020, although appropriate for establishing a molecular census of SCN cells, was not ideal

eN-NWR-0212-21

to detect and quantify expression of low abundance ion channel transcripts such as Trpm4 (or Nalcn).

We did not find these negative results useful, and thus did not include them in the text.

However, along the same lines, we discovered a microarray analysis that reported a 17-fold enrichment

of Trpm4 in the LC, relative to brainstem; and we have now added this information (p. 6).

Additional concerns that should be addressed in the revised manuscript are as follows:

1) The authors focus most of their efforts on oscillations recorded in the presence of TTX, which does not

fully address the issue of how TRPM4 contributes to cell excitability under physiological conditions. The

assumption here is that the subthreshold oscillations seen in the presence of TTX are the subthreshold

drivers of pacemaking under unperturbed conditions. But an alternative view is that the oscillations

measured in the presence of TTX are in fact a meta-phenomenon unique to conditions under which voltage-gated Na+ channels are blocked, and are most apparent in more depolarized voltage ranges. In principle, if subthreshold oscillations drive pacemaking, and TRPM4 channels drive subthreshold oscillations,

then if follows that TRPM4 blockers should diminish pacemaking. Additionally, previous work carried out

in SCN neurons (Jackson et al, J Neurosci 2004) suggests that L-type channel activation during normal

action potential firing is modest, which would in turn limit the contribution of TRPM4. While adding additional data addressing these issues would strengthen the manuscript considerably, the role of TRPM4

during normal spike firing should at least be discussed.

As noted above, we have now performed the requested experiments to assess the effect of inhibiting

TRPM4 channels on spontaneous pacemaker-like firing in LC neurons and SCN neurons. For both cell

groups, we found that 9-phenanthrol (30 uM) reduced baseline firing rate in relatively undisturbed cellattached recording configuration. However, 9-pt did not eliminate spontaneous firing in either cell

group. We now discuss this together with an expanded consideration of previous work examining L-type

and TRPM4 channel contributions to pacemaker-like firing (p. 10-11).

2) The way that the general problem of ionic mechanisms of pacemaking is framed in the Abstract, Introduction and Discussion could be more nuanced. For example, the authors focus on previous work in the

SCN showing that L-type Ca2+ current provides the inward current driving oscillatory activity, and cite

work from Pennartz/2002 and Jackson/2004. In fact, Pennartz and colleagues first implicated a lowthreshold L-type current in providing the depolarizing drive at subthreshold voltages, during the subjective day. While Jackson and colleagues showed that the low-threshold L-type current is there, its primary

role appeared to be driving activation of Ca2+-activated K+ channels, and that a Na+ leak current was

the more important contributor to interspike depolarization. This latter current was later identified as

likely carried by NALCN channels (Flourakis et al, 2015, Cell), which is not cited in this manuscript (and

should be). In light of this, the Discussion could provide a more detailed comparison of the current work

and previous work and tie together possible synergistic interactions between low-threshold L-type current, NALCN and TRPM4 in circadian pacemaking.

Thank you for this suggestion. We have made major changes throughout the manuscript to include additional nuance to our consideration of the different channels that contribute to oscillations. In particular,

a new Discussion section (p. 10-11) notes the influence of Ca2+-activated K+ channels to the effects of Ltype blockers on firing that was previously revealed in SCN neurons. We also highlight the additional

contributions of NALCN channels that have been described in SCN neurons, as mentioned above, as well

as in LC and RTN neurons.

Minor comments

1) The authors should replace ICaN with ICAN in the Abstract, which is the usual abbreviation for Ca2+-

activated nonselective cation current.

eN-NWR-0212-21

This ICAN terminology has now been eliminated.

2) The experiments in this manuscript were conducted at room temperature, which might have strong

effects on intracellular Ca2+ signaling and, consequently, TRPM4 activation. Was there a reason these

recordings were not or could not be carried out at more physiological temperatures?

We find the slices (and cells) stay healthier for longer at room temperature; this is now mentioned in the

Methods (p. 2).

3) Another point of clarification is whether the authors accounted for liquid junction potential in their

current-clamp recordings and in voltage-clamp/oscillation-clamp recordings; this is a particularly important point to clarify given that they used different internal solutions in these experiments.

We now indicate in the Methods section (p. 3) that reported membrane potentials are corrected for the

liquid junction potentials measured with both internal solutions (∼10 mV).

4) Also missing is a discussion of the TRPM4 knockout mouse - particularly whether this model exhibits

any behavioral or physiological defects attributable to LC or SCN function (e.g., Circadian deficits).

We have not found any reports in which behavioral phenotypes attributed to LC or SCN function were

examined in TRPM4 knockout mice. We now point this out in the Discussion (p. 13).

5) The individual data points in Fig 3C, E and F are very large relative to the box-whisker plots, such that

the data points mostly obscure them. This should be adjusted for clarity.

These plots have been modified in the process of converting the presentation and analysis to apply estimation statistics, as requested by the editorial board.
